# 2-[(1*H*-Pyrrol-2-yl)meth­yl]-1*H*-pyrrole

**DOI:** 10.1107/S1600536813028365

**Published:** 2013-10-23

**Authors:** Chong-Hyeak Kim, Yea-Sel Jeon, Vincent Lynch, Jonathan L. Sessler, Kwang-Jin Hwang

**Affiliations:** a Center for Chemical Analysis, Korea Research Institute of Chemical Technology, 141 Gajeongro, Yuseong, Daejeon 305-600, Republic of Korea; b Department of Bio and Chemical Engineering, Hongik University at Sejong, 2639 Sejongro, Jochiwon, Sejong 339-701, Republic of Korea; c Department of Chemistry, The University of Texas, 105 E. 24th St STOP A5300, Austin, TX 78712-1224, USA

## Abstract

In the title compound, C_9_H_10_N_2_, the two pyrrole ring planes are twisted by a dihedral angle of 69.07 (16)° and the C—C—C methane angle is 115.1 (2)°. In the crystal, mol­ecules are connected into layers in the *bc* plane by N—H⋯π inter­actions.

## Related literature

For synthesis of symmetric and non-symmetric porphyrins, see: Shanmugathasan *et al.* (2000 ▶); Bonifazi *et al.* (2005 ▶); Fendt *et al.* (2009 ▶). For their applications as organometallic ligands, see: Ganesan *et al.* (2001 ▶); Gao *et al.* (2004 ▶).
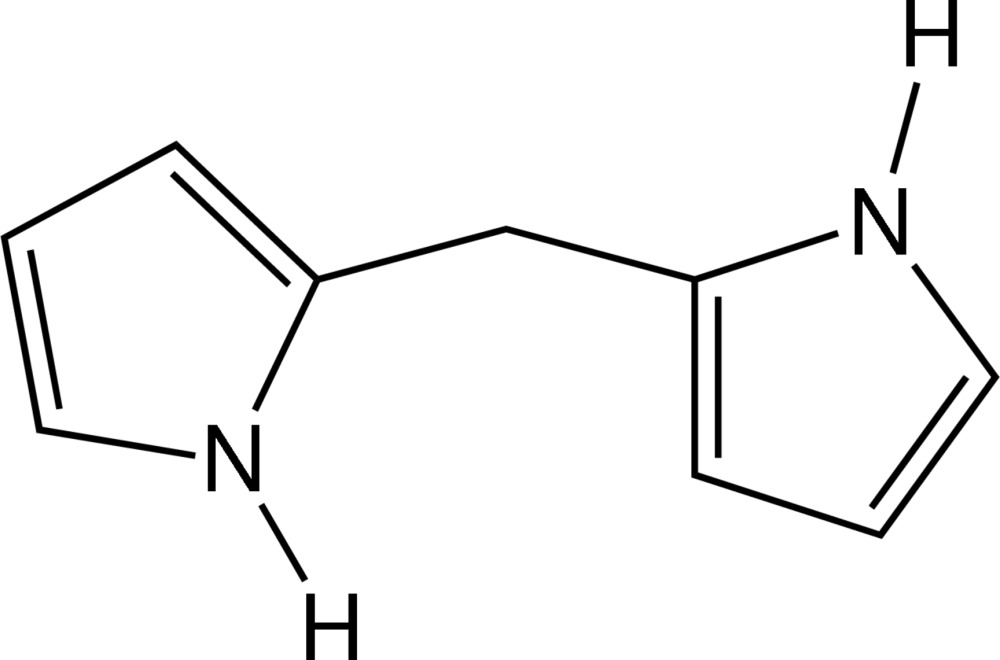


## Experimental

### 

#### Crystal data

C_9_H_10_N_2_
*M* *_r_* = 146.19
Monoclinic, 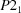

*a* = 6.048 (3) Å
*b* = 7.312 (4) Å
*c* = 9.024 (5) Å
β = 100.78 (1)°
*V* = 392.0 (4) Å^3^
*Z* = 2
Mo *K*α radiation
μ = 0.08 mm^−1^
*T* = 153 K
0.32 × 0.08 × 0.06 mm

#### Data collection

Rigaku SCX-Mini diffractometer with Mercury 2 CCD
Absorption correction: multi-scan (*ABSCOR*; Higashi, 1995 ▶) *T*_min_ = 0.976, *T*_max_ = 0.996
4179 measured reflections
1786 independent reflections
1374 reflections with *I* > 2σ(*I*)
*R*_int_ = 0.063

#### Refinement

*R*[*F* ^2^ > 2σ(*F* ^2^)] = 0.057
*wR*(*F* ^2^) = 0.132
*S* = 1.05
1786 reflections
100 parameters
61 restraints
H-atom parameters constrained
Δρ_max_ = 0.19 e Å^−3^
Δρ_min_ = −0.23 e Å^−3^

### 

Data collection: *CrystalClear* (Molecular Structure Corporation & Rigaku, 2008 ▶); cell refinement: *CrystalClear*; data reduction: *CrystalClear*; program(s) used to solve structure: *SIR97* (Altomare *et al.*, 1999 ▶); program(s) used to refine structure: *SHELXTL/PC* (Sheldrick, 2008 ▶); molecular graphics: *SHELXTL/PC*; software used to prepare material for publication: *SHELXL97* (Sheldrick, 2008 ▶).

## Supplementary Material

Crystal structure: contains datablock(s) hkj, I. DOI: 10.1107/S1600536813028365/tk5264sup1.cif

Structure factors: contains datablock(s) I. DOI: 10.1107/S1600536813028365/tk5264Isup2.hkl

Supplementary material file. DOI: 10.1107/S1600536813028365/tk5264Isup3.cml

Additional supplementary materials:  crystallographic information; 3D view; checkCIF report

## Figures and Tables

**Table 1:** Hydrogen-bond geometry (Å, °) *Cg*1 and *Cg*2 are the centroids of the N1/C1–C4 and N2/C6–C9 rings, respectively.

*D*—H⋯*A*	*D*—H	H⋯*A*	*D*⋯*A*	*D*—H⋯*A*
N1—H1*N*⋯*Cg*1^i^	0.88	2.53	3.357 (3)	156
N2—H2*N*⋯*Cg*2^ii^	0.88	2.53	3.363 (3)	159

Symmetry codes: (i) 
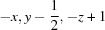
; (ii) 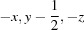.

**Fig. 1.**
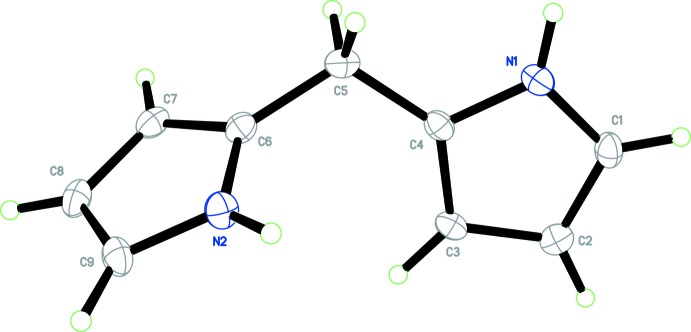
The molecular structure of the title compound with 25% probability displacement ellipsoids.

## supplementary crystallographic information

### 1. Comment

Dipyrromethane (DPM) derivatives have been used as key intermediates in the synthesis of symmetric and non-symmetric porphyrins (Shanmugathasan *et al.*, 2000; Bonifazi *et al.*, 2005; Fendt *et al.*, 2009) and also used as organometallic ligands (Ganesan *et al.*, 2001; Gao *et al.*, 2004). DPMs are typically electron rich and prone to oxidation; this is particularly true in the case of unsubstituted DPMs, which benefit from oxygen-free conditions for isolation and long-term storage. Low temperatures are also beneficial. This sensitivity has made it difficult to obtain unsubstituted dipyrromethanes in the form of X-ray diffraction-grade crystals. Here, we report the crystal structure of 2-(1*H*-pyrrol-2-ylmethyl)-1*H*-pyrrole that in crystalline form is stable in air under ambient conditions. The molecular structure of the title compound is shown in Fig. 1. The configuration of two pyrrole ring planes are approximately perpendicular to each other, with the C4—C5—C6 methane angle of 115.1 (2)°.

### 2. Experimental

For the synthesis of DPM, the solution of paraformaldehyde (0.9 g, 29.97 mmol) in pyrrole (110 ml, 1.58 mol) with InCl_3_ (0.3 g, 1.42 mmol) was stirred for 1 h at 70 °C under nitrogen atmosphere. After addition of NaOH (5 pellets), the reaction solution was stirred for 1 h at room temperature and then concentrated under vacuum (20 mmHg) at 70 °C. To the reaction mixture was poured 1 N NaOH solution (100 ml) and ethyl acetate (100 ml), then the organic layer was dried (Na_2_SO_4_), and distilled to afford DPM (4.37 g, 50% yield) as a dark brown syrup. The crystals of the title compound suitable for X-ray analysis were collected in the form of long needles from the pyrrole-rich distillate after being stored in a freezer for few days.

### 3. Refinement

H atoms were placed in calculated positions using a riding model with N—H = 0.88 Å and C—H = 0.95 and 0.99 Å for pyrrole and methane H, respectively, and *U*_iso_(H) = 1.2 *U*_eq_(C,*N*).

### Crystal data

**Table:**

C_9_H_10_N_2_	*F*(000) = 156
*M**_r_* = 146.19	*D*_x_ = 1.238 Mg m^−^^3^
Monoclinic, *P*2_1_	Mo *K*α radiation, λ = 0.71075 Å
Hall symbol: P 2yb	Cell parameters from 4189 reflections
*a* = 6.048 (3) Å	θ = 3.0–27.5°
*b* = 7.312 (4) Å	µ = 0.08 mm^−^^1^
*c* = 9.024 (5) Å	*T* = 153 K
β = 100.78 (1)°	Needle, colourless
*V* = 392.0 (4) Å^3^	0.32 × 0.08 × 0.06 mm
*Z* = 2	

### Data collection

**Table:**

Rigaku SCX-Mini with Mercury 2 CCD diffractometer	1786 independent reflections
Radiation source: fine-focus sealed tube	1374 reflections with *I* > 2σ(*I*)
Graphite monochromator	*R*_int_ = 0.063
ω scans	θ_max_ = 27.5°, θ_min_ = 3.4°
Absorption correction: multi-scan (*ABSCOR*; Higashi, 1995)	*h* = −7→7
*T*_min_ = 0.976, *T*_max_ = 0.996	*k* = −9→9
4179 measured reflections	*l* = −11→11

### Refinement

**Table:**

Refinement on *F*^2^	Secondary atom site location: difference Fourier map
Least-squares matrix: full	Hydrogen site location: inferred from neighbouring sites
*R*[*F*^2^ > 2σ(*F*^2^)] = 0.057	H-atom parameters constrained
*wR*(*F*^2^) = 0.132	*w* = 1/[σ^2^(*F*_o_^2^) + (0.049*P*)^2^] where *P* = (*F*_o_^2^ + 2*F*_c_^2^)/3
*S* = 1.05	(Δ/σ)_max_ = 0.001
1786 reflections	Δρ_max_ = 0.19 e Å^−^^3^
100 parameters	Δρ_min_ = −0.23 e Å^−^^3^
61 restraints	Absolute structure: nd
Primary atom site location: structure-invariant direct methods	

### Special details

**Table:**

Geometry. All e.s.d.'s (except the e.s.d. in the dihedral angle between two l.s. planes) are estimated using the full covariance matrix. The cell e.s.d.'s are taken into account individually in the estimation of e.s.d.'s in distances, angles and torsion angles; correlations between e.s.d.'s in cell parameters are only used when they are defined by crystal symmetry. An approximate (isotropic) treatment of cell e.s.d.'s is used for estimating e.s.d.'s involving l.s. planes.
Refinement. Refinement of *F*^2^ against ALL reflections. The weighted *R*-factor *wR* and goodness of fit *S* are based on *F*^2^, conventional *R*-factors *R* are based on *F*, with *F* set to zero for negative *F*^2^. The threshold expression of *F*^2^ > σ(*F*^2^) is used only for calculating *R*-factors(gt) *etc*. and is not relevant to the choice of reflections for refinement. *R*-factors based on *F*^2^ are statistically about twice as large as those based on *F*, and *R*- factors based on ALL data will be even larger. The direction of the twofold screw axis could not be reliably determined.

### Fractional atomic coordinates and isotropic or equivalent isotropic displacement parameters (Å^2^)

**Table:**

	*x*	*y*	*z*	*U*_iso_*/*U*_eq_	
C1	0.2619 (4)	−0.0035 (4)	0.5615 (3)	0.0353 (6)	
H1	0.3300	−0.0409	0.6603	0.042*	
C2	0.3488 (4)	0.1182 (4)	0.4732 (3)	0.0331 (6)	
H2	0.4887	0.1802	0.4991	0.040*	
C3	0.1930 (4)	0.1352 (3)	0.3367 (3)	0.0298 (6)	
H3	0.2088	0.2109	0.2538	0.036*	
C4	0.0136 (4)	0.0222 (4)	0.3451 (2)	0.0284 (5)	
C5	−0.2014 (4)	−0.0146 (4)	0.2387 (2)	0.0348 (6)	
H5A	−0.3267	0.0374	0.2818	0.042*	
H5B	−0.2242	−0.1486	0.2310	0.042*	
C6	−0.2144 (4)	0.0606 (3)	0.0837 (3)	0.0301 (6)	
C7	−0.3568 (4)	0.1863 (4)	0.0037 (3)	0.0342 (6)	
H7	−0.4721	0.2516	0.0396	0.041*	
C8	−0.3029 (4)	0.2019 (4)	−0.1402 (3)	0.0362 (6)	
H8	−0.3761	0.2785	−0.2195	0.043*	
C9	−0.1268 (4)	0.0876 (4)	−0.1462 (2)	0.0367 (6)	
H9	−0.0530	0.0711	−0.2294	0.044*	
N1	0.0597 (4)	−0.0620 (3)	0.4825 (2)	0.0344 (6)	
H1N	−0.0283	−0.1423	0.5153	0.041*	
N2	−0.0764 (3)	0.0011 (3)	−0.0094 (2)	0.0336 (5)	
H2N	0.0302	−0.0813	0.0148	0.040*	

### Atomic displacement parameters (Å^2^)

**Table:**

	*U*^11^	*U*^22^	*U*^33^	*U*^12^	*U*^13^	*U*^23^
C1	0.0380 (14)	0.0427 (15)	0.0244 (12)	0.0079 (13)	0.0037 (11)	−0.0002 (12)
C2	0.0322 (13)	0.0322 (14)	0.0356 (13)	−0.0001 (11)	0.0082 (11)	−0.0078 (11)
C3	0.0366 (13)	0.0265 (13)	0.0292 (12)	−0.0002 (10)	0.0135 (11)	0.0000 (10)
C4	0.0349 (12)	0.0269 (13)	0.0249 (11)	0.0027 (10)	0.0096 (10)	0.0001 (9)
C5	0.0311 (12)	0.0358 (14)	0.0388 (14)	−0.0030 (11)	0.0103 (11)	0.0009 (11)
C6	0.0281 (12)	0.0290 (14)	0.0322 (13)	−0.0035 (10)	0.0030 (10)	−0.0048 (10)
C7	0.0248 (13)	0.0328 (14)	0.0438 (15)	0.0010 (11)	0.0031 (11)	0.0001 (11)
C8	0.0343 (14)	0.0296 (14)	0.0394 (14)	−0.0002 (11)	−0.0070 (12)	0.0034 (11)
C9	0.0459 (14)	0.0375 (16)	0.0248 (13)	−0.0032 (13)	0.0016 (11)	−0.0026 (11)
N1	0.0388 (12)	0.0334 (13)	0.0323 (11)	−0.0030 (10)	0.0100 (10)	0.0038 (9)
N2	0.0371 (11)	0.0287 (11)	0.0345 (11)	0.0064 (10)	0.0058 (9)	0.0006 (9)

### Geometric parameters (Å, º)

**Table:**

C1—C2	1.363 (4)	C5—H5B	0.9900
C1—N1	1.364 (3)	C6—N2	1.361 (3)
C1—H1	0.9500	C6—C7	1.370 (3)
C2—C3	1.410 (3)	C7—C8	1.402 (3)
C2—H2	0.9500	C7—H7	0.9500
C3—C4	1.377 (3)	C8—C9	1.363 (3)
C3—H3	0.9500	C8—H8	0.9500
C4—N1	1.365 (3)	C9—N2	1.369 (3)
C4—C5	1.490 (3)	C9—H9	0.9500
C5—C6	1.491 (3)	N1—H1N	0.8800
C5—H5A	0.9900	N2—H2N	0.8800
			
C2—C1—N1	107.8 (2)	N2—C6—C7	106.7 (2)
C2—C1—H1	126.1	N2—C6—C5	122.0 (2)
N1—C1—H1	126.1	C7—C6—C5	131.2 (2)
C1—C2—C3	107.5 (2)	C6—C7—C8	108.1 (2)
C1—C2—H2	126.3	C6—C7—H7	125.9
C3—C2—H2	126.3	C8—C7—H7	125.9
C4—C3—C2	107.7 (2)	C9—C8—C7	107.8 (2)
C4—C3—H3	126.2	C9—C8—H8	126.1
C2—C3—H3	126.2	C7—C8—H8	126.1
N1—C4—C3	107.0 (2)	C8—C9—N2	107.0 (2)
N1—C4—C5	120.6 (2)	C8—C9—H9	126.5
C3—C4—C5	132.4 (2)	N2—C9—H9	126.5
C4—C5—C6	115.1 (2)	C1—N1—C4	110.1 (2)
C4—C5—H5A	108.5	C1—N1—H1N	124.9
C6—C5—H5A	108.5	C4—N1—H1N	124.9
C4—C5—H5B	108.5	C6—N2—C9	110.5 (2)
C6—C5—H5B	108.5	C6—N2—H2N	124.8
H5A—C5—H5B	107.5	C9—N2—H2N	124.8
			
N1—C1—C2—C3	0.5 (3)	C5—C6—C7—C8	−177.2 (2)
C1—C2—C3—C4	−0.1 (3)	C6—C7—C8—C9	−0.7 (3)
C2—C3—C4—N1	−0.4 (3)	C7—C8—C9—N2	1.1 (3)
C2—C3—C4—C5	178.4 (3)	C2—C1—N1—C4	−0.8 (3)
N1—C4—C5—C6	−170.1 (2)	C3—C4—N1—C1	0.7 (3)
C3—C4—C5—C6	11.2 (4)	C5—C4—N1—C1	−178.3 (2)
C4—C5—C6—N2	64.4 (3)	C7—C6—N2—C9	0.6 (3)
C4—C5—C6—C7	−118.6 (3)	C5—C6—N2—C9	178.2 (2)
N2—C6—C7—C8	0.1 (3)	C8—C9—N2—C6	−1.0 (3)

### Hydrogen-bond geometry (Å, º)

Cg1 and Cg2 are the centroids of the N1/C1–C4 and N2/C6–C9 rings, respectively.

**Table:**

*D*—H···*A*	*D*—H	H···*A*	*D*···*A*	*D*—H···*A*
N1—H1*N*···*Cg*1^i^	0.88	2.53	3.357 (3)	156
N2—H2*N*···*Cg*2^ii^	0.88	2.53	3.363 (3)	159

Symmetry codes: (i) −*x*, *y*−1/2, −*z*+1; (ii) −*x*, *y*−1/2, −*z*.
